# Beneficial Effects of *Teucrium polium* and Metformin on Diabetes-Induced Memory Impairments and Brain Tissue Oxidative Damage in Rats

**DOI:** 10.1155/2015/493729

**Published:** 2015-02-24

**Authors:** S. Mojtaba Mousavi, Saeed Niazmand, Mahmoud Hosseini, Zarha Hassanzadeh, Hamid Reza Sadeghnia, Farzaneh Vafaee, Zakieh Keshavarzi

**Affiliations:** ^1^Neurogenic Inflammation Research Center, School of Medicine, Mashhad University of Medical Sciences, Mashhad 9177948564, Iran; ^2^Neurocognitive Research Center, School of Medicine, Mashhad University of Medical Sciences, Mashhad 9177948564, Iran; ^3^Pharmacological Research Center of Medicinal Plants, School of Medicine, Mashhad University of Medical Sciences, Mashhad 9177948564, Iran; ^4^Department of Physiology, School of Medicine, North Khorasan University of Medical Sciences, Bojnourd, Iran

## Abstract

*Objective.* The effects of hydroalcoholic extract of *Teucrium polium* and metformin on diabetes-induced memory impairment and brain tissues oxidative damage were investigated. *Methods.* The rats were divided into: (1) Control, (2) Diabetic, (3) Diabetic-Extract 100 (Dia-Ext 100), (4) Diabetic-Extract 200 (Dia-Ext 200), (5) Diabetic-Extract 400 (Dia-Ext 400), and (6) Diabetic-Metformin (Dia-Met). Groups 3–6 were treated by 100, 200, and 400 mg/kg of the extract or metformin, respectively, for 6 weeks (orally). *Results*. In passive avoidance test, the latency to enter the dark compartment in Diabetic group was lower than that of Control group (*P* < 0.01). In Dia-Ext 100, Dia-Ext 200, and Dia-Ext 400 and Metformin groups, the latencies were higher than those of Diabetic group (*P* < 0.01). Lipid peroxides levels (reported as malondialdehyde, MDA, concentration) in the brain of Diabetic group were higher than Control (*P* < 0.001). Treatment by all doses of the extract and metformin decreased the MDA concentration (*P* < 0.01). *Conclusions.* The results of present study showed that metformin and the hydroalcoholic extract of* Teucrium polium* prevent diabetes-induced memory deficits in rats. Protection against brain tissues oxidative damage might have a role in the beneficial effects of the extract and metformin.

## 1. Introduction

Diabetes is a growing problem throughout the world. The global prevalence of established diabetes is estimated to be 2.8% in 2000 and is predicted to be 4.4% by 2030 [1]. Diabetes generates a variety of functional and structural disturbances in the different organs especially central and peripheral nervous systems [2, 3]. Moreover, a wide range of learning and memory impairments have been observed in adults with diabetes mellitus [4–6]. Electrophysiological and structural abnormalities of the brain of diabetic subjects have also been reported [3]. Diabetes also conceivably leads to cognitive impairments through chronic hyperglycemia [7].

It has been well documented that oxidative stress plays a pivotal role in the development of diabetes complications, including learning and memory impairments [8, 9]. Enhanced formation of oxygen free radicals occurs in tissues during hyperglycemia. These oxidant radicals contribute to increased neuronal death in many brain regions such as the hippocampus, through protein oxidation, DNA damage, and peroxidation of membrane lipids [10].

The medicinal use of plants dates back to ancient times.* Teucrium polium* L. (Lamiaceae) has been used for over 2000 years in traditional medicine due to its diuretic, diaphoretic, tonic, antipyretic, antispasmodic, and cholagogic properties [11]. In addition, the plant possesses hypoglycemic, hypolipidemic, insulinotropic, antioxidant, antinociceptive, and anti-inflammatory properties [12–15]. It also affects cardiovascular system [16], reduces body weight [17], protects against acetaminophen-induced hepatotoxicity [18–21], and improves cognitive deficits [22].

Considering these beneficial effects of* Teucrium polium*, the objective of this study was to investigate the effects of long-term oral administration of* Teucrium polium* and metformin on learning and memory impairments in diabetic rats using a passive avoidance test. Markers of oxidative stress, mainly, lipid peroxides levels (reported as malondialdehyde, MDA) and total thiol (SH) content, were also measured in cortical and hippocampal regions.

## 2. Materials and Methods

### 2.1. Preparing the Plant Extract

*Teucrium polium* was collected from Nishabur City, Khorasan Province, Iran, and identified by botanists in Ferdowsi University of Mashhad, Iran, and a voucher specimen was deposited. The plants were then dried at room temperature. To prepare the hydroalcoholic extract, 50 g of the chopped and dried aerial parts of the plant was soaked in ethanol (50%) for 48 h and filtered through paper filter. The extract was then dried with rotary vacuum evaporator.

### 2.2. Animals and the Experimental Protocol

Male Wistar rats (10 weeks old and weighing 250 ± 20 g) were kept at 22 ± 2°C and 12 h light/dark cycle at 7:00 a.m. They were randomly divided into six groups and treated according to the experimental protocol:

(1) Control, (2) Diabetic, (3) Diabetic-Extract 100 (Dia-Ext 100), (4) Diabetic-Extract 200 (Dia-Ext 200), (5) Diabetic-Extract 400 (Dia-Ext 400), and (6) Diabetic-Metformin (Dia-Met). Diabetes was induced in groups 2–6 by a single intraperitoneal (i.p.) injection of streptozotocin (55 mg/kg) [23]. The animals of groups 3–5 were orally treated by 100, 200, and 400 mg/kg of the extract for 6 weeks. The animals of Dia-Met group were treated by metformin (300 mg/kg) [24]. The treatments by the extract or metformin were started 72 hours after STZ injection. The extract and metformin were dissolved in saline. Only the animals with serum glucose higher than 250 mg/dL at 72 h after streptozotocin injection were included in the study. All efforts were made to maintain the animals in good general health, in accordance with the European Communities Council Directive (2010/63/UE). Animal handling and all related procedures were confirmed by Mashhad University of Medical Sciences, Ethical Committee.

### 2.3. Passive Avoidance Test

The passive avoidance learning test based on negative reinforcement was carried out. The apparatus had a grid floor and comprised two compartments: one dark and the other one lighted, with a small gate which connected these two parts. This test is performed with the knowledge that rats have a native preference for the dark environment. Before beginning the training sessions, the animals were familiarized with the apparatus for two successive days (5 min per day). On the ensuing day, they were placed in the lighted compartment and the time latency for entering the dark compartment and the time spent in dark and light compartments were noted down. During the training phase, the animals were located in the lighted compartment while they were facing the walls and away from the gate and they received an electric shock (1.5 mA, 2 s duration) when they were entered the dark part. The animals were then returned to their cages. In retention or test phase, which was carried out at one hour and twenty-four hours after the training session, the rats were placed in the light compartment, and time latency to enter the dark compartment and the time spent by the animals in the dark and light compartments were recorded [25]. All behavioral tests were conducted between 16:00 and 18:00 o'clock.

### 2.4. Biochemical Assessments

After the behavioral study, blood samples were collected for determination of serum glucose level, the animals were then sacrificed, and the brains were removed and dissected on an ice-cold surface and conserved for biochemical measurements.

Total SH groups were measured using DTNB (2,2′-dinitro-5,5′-dithiodibenzoic acid) as the reagent. This reagent reacts with the SH groups to produce a yellow colored complex which has a peak absorbance at 412 nm. Briefly, 1 mL Tris-EDTA (ethylenediaminetetraacetic acid) buffer (pH = 8.6) was added to 50 *μ*L brain homogenate in 1 mL cuvettes and sample absorbance was read at 412 nm against Tris-EDTA buffer alone (*A*_1_). Then 20 *μ*L DTNB reagents (10 mM in methanol) were added to the mixture and after 15 min (at laboratory temperature) the sample absorbance was read again (*A*_2_). The absorbance of DTNB reagent was also read as a blank (*B*). Total thiol concentration (mM) was calculated from the following equation [26–28]:

(1) Total  thiol  concentration  mM  =A2−A1−B×1.070.05×13.6.

Malondialdehyde (MDA) levels, as an index of lipid peroxidation, were also measured. MDA reacts with thiobarbituric acid (TBA) as a thiobarbituric acid reactive substance (TBARS) to produce a pink colored complex which has peak absorbance at 535 nm. 2 mL of TBA/TCA (trichloroacetic acid)/HCL (hydrochloric acid) reagent was added to 1 mL of homogenate and the solution was heated in a water bath for 40 min. After cooling, the solution was centrifuged at 1000 g for 10 min. The absorbance was measured at 535 nm. The MDA concentration was calculated as follows: *C*(m) = Absorbance/(1.65 × 10^5^) [27, 28].

### 2.5. Statistical Analysis

The data were expressed as mean ± SEM. One-way ANOVA was run followed by Tukey's post hoc test comparisons test (SPSS 11.5 software). The criterion for the statistical significance was *P* < 0.05.

## 3. Results

### 3.1. Passive Avoidance Test

**Figure 1:**
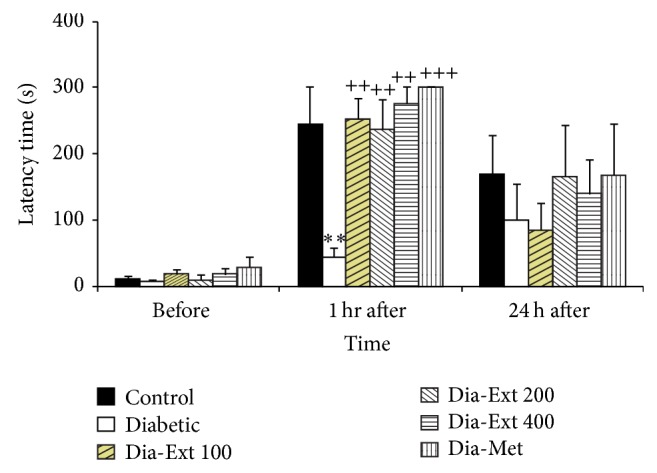
Comparison of time latency for entering the dark compartment before and at 1 and 24 h after receiving the shock in the experimental groups. Data are presented as mean ± SEM (*n* = 10 in each group). ^**^ *P* < 0.01 in comparison with Control group and ^++^ *P* < 0.01 and ^+++^ *P* < 0.001 in comparison with Diabetic group.

In the Diabetic group, the time latency for entering the dark compartment was lower than that of the Control group, 1 h after shock (Figure 1, *P* < 0.01). The treatment of the animals by 100, 200, and 400 mg/kg of* Teucrium polium* extract significantly increased the time latency for entering the dark compartment at 1 h after receiving a shock (Figure 1, *P* < 0.01). Treatment by metformin also increased the time latency for entering the dark compartment at 1 h after receiving a shock. There were no significant differences between groups at 24 hours after shock (Figure 1).

**Figure 2:**
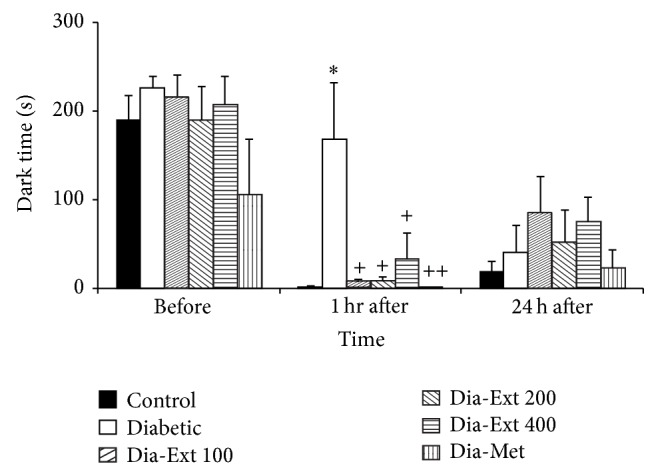
Comparison of the total time spent in the dark compartment before and at 1 and 24 h after receiving the shock in the experimental groups. Data are presented as mean ± SEM (*n* = 10 in each group). ^*^ *P* < 0.05 in comparison with Control group and ^+^ *P* < 0.05 and ^++^ *P* < 0.01 in comparison with Diabetic group.

One hour after receiving the shock, the total time spent in the dark compartment by the animals of the Diabetic group was more than that of the Control group (Figure 2, *P* < 0.05). Treatment by all three doses of the extract decreased the total time spent in dark compartment at 1 hour after receiving the shock (Figure 2, *P* < 0.05). The total time spent in the dark compartment by the animals of Dia-Met group was lower than that of the Diabetic group (Figure 2, *P* < 0.01).

**Figure 3:**
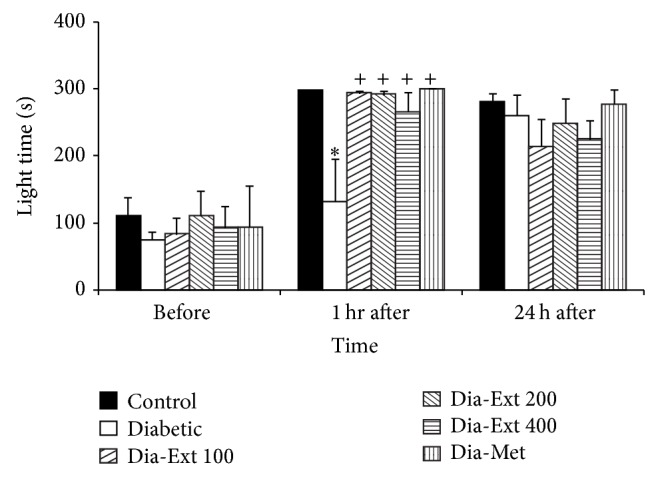
Comparison of the total time spent in the light compartment before and at 1 and 24 h after receiving the shock in the experimental groups. Data are presented as mean ± SEM (*n* = 10 in each group). ^*^ *P* < 0.05 in comparison with Control group and ^+^ *P* < 0.05 in comparison with Diabetic group.

The results also showed that the time spent in the light compartment by the animals of the Diabetic group was lower than that of the Control group at 1 h after receiving the shock (Figure 3, *P* < 0.05); however, there was no significant difference at 24 h after the shock. Treatment of the animals by 100–400 mg/kg of the extract and metformin increased the time spent in the light compartment when the animals were examined at 1 h after receiving a shock (Figure 3). There were no significant differences between groups at 24 h after the shock.

### 3.2. Biochemical Assessment Results

**Figure 4:**
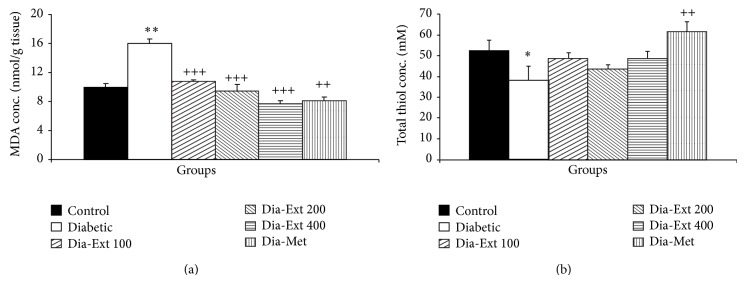
The MDA concentrations (a) and total thiol concentrations (b) in hippocampal tissues of 6 groups. Data are shown as mean ± SEM of 10 animals per group. ^*^ *P* < 0.05 and ^***^ *P* < 0.001 in comparison with Control group and ^++^ *P* < 0.01 and ^+++^ *P* < 0.001 in comparison with Diabetic group.

MDA concentration in hippocampal tissues of the Diabetic group was higher than Control ones (Figure 4(a); *P* < 0.001). Pretreatment of the animals by 100, 200, and 400 mg/kg of the extract decreased the MDA concentration in the hippocampal tissues in the Diabetic group (Figure 4(a); all *P* < 0.001). Treatment of diabetic rats by metformin also reduced MDA concentration in hippocampal tissues (Figure 4(a); all *P* < 0.001).

The total thiol concentration in hippocampal tissues of the Diabetic group was significantly lower than Control animals (Figure 4(b); *P* < 0.05). In Dia-Met group, the hippocampal total thiol concentrations were significantly higher than that of the Diabetic group (Figure 4(b); *P* < 0.01); however, treatment with the extract with doses of 100–400 mg/kg was not effective in reducing total thiol concentrations.

**Figure 5:**
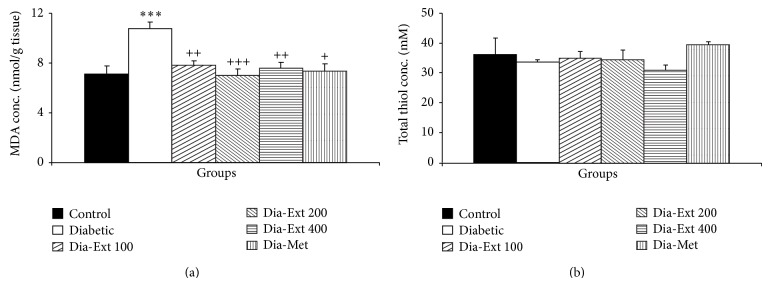
The MDA concentrations (a) and total thiol concentrations (b) in cortical tissues of 6 groups. Data are shown as mean ± SEM of 10 animals per group. ^***^ *P* < 0.001 in comparison with Control group and ^++^ *P* < 0.01 and ^+++^ *P* < 0.001 in comparison with Diabetic group.

The results also showed that cortical MDA concentration in Diabetic group was significantly higher than Control group (Figure 5(a); *P* < 0.001). In cortical tissues of Dia-Ext 100, Dia-Ext 200, and Dia-Ext 400 groups, MDA concentrations were lower than that of Diabetic group (Figure 5(a); *P* < 0.01 and *P* < 0.001). The animals of Dia-Met group also had a lower MDA concentration in cortical tissues when compared with Diabetic group (Figure 5(a); *P* < 0.01).

As shown in Figure 5(a), the total thiol concentration in cortical tissues of the Diabetic group was lower than from Control group (Figure 5(b)). Administration of the extract did not affect the total thiol concentration (Figure 5(b)). Metformin nonsignificantly increased the total thiol concentration in cortical tissues.

**Figure 6:**
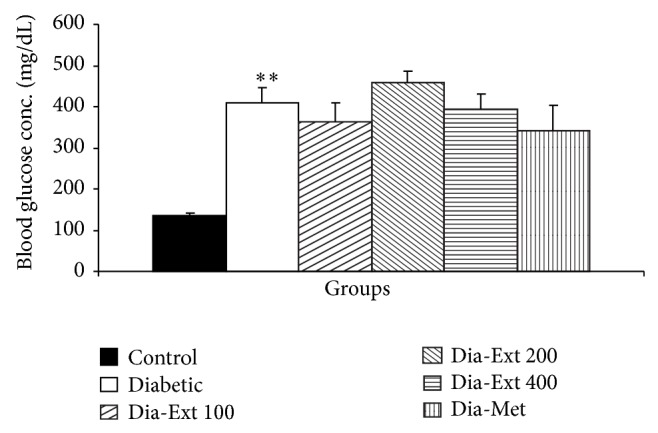
The blood glucose concentrations of 6 groups. Data are shown as mean ± SEM of 10 animals per group. ^**^ *P* < 0.01 in comparison with Control group.

Blood glucose concentration in the Diabetic group was significantly higher than Control group (Figure 6; *P* < 0.01). Administration of the extract did not affect the glucose concentration (Figure 6). Metformin nonsignificantly reduced the blood glucose concentration.

## 4. Discussion

The present study demonstrated that chronic hyperglycemia in diabetic rats significantly impaired learning and memory. Treatment with the different doses of* Teucrium polium* for 6 weeks improved the deleterious effects of diabetes on learning and memory. Consistent with our results, learning deficits have been shown in streptozotocin-induced diabetic rats in Morris water maze [29]. Evidence is accumulating that people with diabetes mellitus are at risk of developing cognitive impairments [30, 31]. It has also been demonstrated that acute hyperglycemia in people with type 2 diabetes significantly impaired speed of information processing, working memory, and some aspects of attention [31]. This deficiency in learning and memory has been suggested to be associated with the changes in hippocampal synaptic plasticity [9, 32]. It has also been suggested that oxidative stress contributes to the learning and memory deficits during hyperglycemia [33]. In the current experiment, it was also found that MDA levels, as an index of lipid peroxidation, were increased in the hippocampal and cortical tissues of diabetic rats. Total thiol contents were also decreased in brain tissues of diabetic rats. These results confirmed the previous reports that streptozotocin-induced diabetes was accompanied by an increased generation of reactive species. It is also suggested that the antioxidant compounds may have beneficial effects against the complications of diabetes mellitus [34–36].

The results of the present study showed that metformin improved learning and memory; it also reduced MDA concentration and increased total thiol groups in brain tissues of diabetic rats. In this study, we present evidence that metformin exerts an antioxidative effect,* in vivo.* This finding is in accordance with other studies which have shown that metformin treatment has antioxidant properties in STZ-diabetic rats [37, 38]. Similar observations have reported that metformin reduces oxidative stress in various animal models [39, 40]. It has been previously suggested that metformin also has antioxidant activity which is independent of its effect on insulin activity [39]. In the current study, metformin had no significant effect on blood glucose levels in diabetic rats. It has been previously shown that metformin was not able to reduce the plasma levels of glucose in animal models with a high level of glucose similar to the present study [37]. In the present study, a lower dose of metformin (300 mg/kg) was used and it reduced blood glucose concentrations but the effect was not significant. It is reported that metformin reduces blood glucose level at much higher doses of 450–500 mg/kg in STZ-diabetic rats [41]. The results of human studies have also shown that metformin reduces the plasma glucose concentrations by approximately 20 to 30 percent and it has been suggested to be used in combination with other drugs [42]. Regarding the results of present study, it seems that the effects of metformin on learning and memory which were seen in the present study are probably independent of the effect on blood glucose level. Recently, metformin has been reported to improve microvascular function in type 2 diabetic models without improving hyperglycemia [43]. Sartoretto et al. (2005) have also reported that metformin increases nitric oxide activity in diabetic rats [43]. Nitric oxide is rapidly inactivated by O_2_^−^ and it has been reported that enhanced formation of O_2_^−^ radical may be involved in the accelerated breakdown of nitric oxide [44]. Moreover, it has been shown that rapid destruction of nitric oxide occurs in diabetic rats [45]. The protective effect of metformin against oxidative stress may prevent the breakdown of nitric oxide, which may improve learning and memory tasks. Nitric oxide as a gaseous neurotransmitter has been shown to have an important role in learning and memory [46]. It has been previously reported that, when administered for 4 weeks, metformin lowered blood glucose level; however, these plasma glucose levels were still significantly higher than those of Control rats [38]. It has also been reported that metformin reduces vascular O_2_^−^ anion in aortic rings and the amounts of protein carbonyl compounds in serum of diabetic high-fat fed rats [38]. Several studies have reported the inhibitory effect of metformin on oxidative stress under various conditions:* in vitro* in either hyperglycemic or hyperlipidemic environments and* in vivo* [47–49]. Recently, it was reported that metformin may also diminish oxidative stress-related DNA damage [50]. Also, metformin treatment diminished the amounts of serum oxidized proteins and tissue lipid peroxides in diabetic rats [51]. The reduction of oxidative stress by metformin may partly be due to inhibition of glycation, a process that directly causes free-radical production. A recent study suggests that the intracellular antioxidant properties of metformin may result in the inhibition of both the advanced glycation end-products (AGEs) receptor and the lectin-like oxidized receptor [52].

In the present study,* Teucrium polium* hydroalcoholic extract prevented learning and memory impairments in diabetic rats. In accordance with the present study, it has been reported that* Teucrium polium* protects against memory impairments in scopolamine- and diabetes-induced memory impairment models [22, 53].* Teucrium polium* has been reported to contain diterpenes and flavonoids [54]. The notable activity of* Teucrium polium* in memory augmentation could be related to its terpenic and flavonoid compounds [55]. The hypoglycemic effects of* Teucrium polium* have been previously reported [12, 14]. In the present study the extract did not significantly reduce blood glucose level. Therefore, it seems that the beneficial effect of the extract on learning and memory impairments, which was seen in the present study, is not related to the blood glucose-lowering effect. In contrast to some reports which have suggested the blood glucose-lowering effects of* Teucrium polium* [14], there are also some reports which fail to confirm these effects [12, 56]. Gharaibeh et al. reported that a decoction extract of the plant lowered the blood glucose by 20% similar to what was seen in the present study [12]. In another study, a percolated extract of the plant was not able to reduce the blood sugar in type II diabetic patients [56]. The discrepancies might be due to the type of extract. In the present study it was indicated that all three doses of* Teucrium polium* extract significantly reduced lipid peroxidation in the hippocampus and cerebral cortex. These marked protective effects of* Teucrium polium* against oxidative stress observed in this study are consistent with the previously published reports [57]. In this regard, a methanolic extract of* Teucrium polium* protected red blood cells against lipid peroxidation induced by hydrogen peroxide [58]. In another study, Panovska and coworkers [59] demonstrated that the extracts of* Teucrium polium* prepared using different organic solvents (diethyl ether, ethyl acetate, and n-butanol) were effective inhibitors of *β*-carotene oxidation. In another study, it was shown that the extracts prepared from* Teucrium polium* suppressed lipid peroxidation* in vitro* [60]. It is suggested that this high antioxidant activity is due to the phenolic compounds detected in this herb such as hydroxybenzoic acid derivatives, caffeic acid, ferulic acid, and flavonoid derivatives such as luteolin and quercetin [61]. It has been reported that* Teucrium polium* (200 and 400 mg/kg) prevented the deleterious effects of diabetes on passive avoidance memory, but 100 mg/kg of* Teucrium polium* did not have any positive effect on the diabetes-induced memory deficits [22]. In the current study, memory deficits were prevented by all three doses of* Teucrium polium* (100, 200, and 400 mg/kg). On the other hand, we also indicated that all three doses of* Teucrium polium* significantly reduced lipid peroxidation in the hippocampus and cerebral cortex suggesting that protection against oxidative stress was probably involved in the learning and memory enhancing properties of the extract. The high dose of the extract also increased total thiol concentration in hippocampal tissues. In contrast to the beneficial effects of* Teucrium polium* which were mentioned and were seen in the present study, hepatotoxic and nephrotoxic effects of the plant have also been reported [62] and should be considered before reaching a final conclusion about the utility of this plant.

The role of oxidative stress in complications of diabetes has been studied extensively in experimental diabetes models and diabetic patients [63, 64]. Due to the hyperglycemia associated with diabetes, enhanced formation of reactive oxygen occurs, which contributes to the increased neuronal death by oxidizing proteins, damaging DNA, and augmenting lipid peroxidation [10, 65]. Oxidative damage to the rat synapse in the cerebral cortex and hippocampus has been previously reported to contribute to the deficit of cognitive functions [8, 66]. Therefore, antioxidants might be of general use in the prevention of the neurodegeneration deficits and cognitive impairments associated with diabetes. The present study showed that treatment with* Teucrium polium* prevented the learning and memory deficits associated with STZ-induced diabetes. The antioxidant property of* Teucrium polium* may reduce oxidative damage to the synapses in the hippocampus and cerebral cortex and therefore improve learning and memory deficits [66].

In general, it was found that* Teucrium polium* significantly ameliorated the cognitive impairment in diabetic rats. The exact mechanism of* Teucrium polium* in preventing learning and memory deficits is still in debate. In the current study, both oxidative stress and deficits in learning and memory induced by diabetes were prevented by the treatment with* Teucrium polium* suggesting that oxidative stress was probably involved in the diabetes-induced cognitive deficits which were prevented by* Teucrium polium* extract.
